# Exploring current hypervirulent *Klebsiella pneumoniae* infections: insights into pathogenesis, drug resistance, and vaccine prospects

**DOI:** 10.3389/fmicb.2025.1604763

**Published:** 2025-05-29

**Authors:** Qingjing Wang, Haojie Yu, Xueer Pan, Weichang Huang, Jonathan Lalsiamthara, Saif Ullah, Yongchang Xu, Anwei Lu

**Affiliations:** ^1^Zhejiang Shuren University, Hangzhou, China; ^2^Key Laboratory of Pollution Exposure and Health Intervention of Zhejiang Province, Shulan International Medical College, Zhejiang Shuren University, Hangzhou, China; ^3^Stomatology Hospital, School of Medicine, Zhejiang University, Hangzhou, China; ^4^Department of Molecular Microbiology and Immunology, School of Medicine, Oregon Health & Science University, Portland, OR, United States; ^5^Department of Molecular Genetics and Microbiology, University of New Mexico, Albuquerque, NM, United States; ^6^Hangzhou Key Laboratory of Inflammation and Immunoregulation, Department of Basic Medical Science, School of Medicine, Hangzhou Normal University, Hangzhou, China

**Keywords:** *Klebsiella pneumoniae*, drug resistance, virulence, pathogenesis, vaccine, epidemiology

## Abstract

*Klebsiella pneumoniae* is a significant pathogenic bacterium responsible for a range of infections. The escalating prominence of *K. pneumoniae* in hospital-acquired infections is a deeply alarming trend that demands immediate attention and rigorous intervention. This article provides an up-to-date review of *K. pneumoniae*’s virulence factors, pathogenesis, and the mechanism driving drug resistance. It also explores the potential for safe and effective vaccine developments, vital for preventing and controlling these diseases. Furthermore, we summarize the epidemiological characteristics of classical and hypervirulent *K. pneumoniae* infections, providing an overview of drug-resistance *K. pneumoniae* emergence, transmission, and prevalence.

## 1 Introduction

*Klebsiella pneumoniae*, is a Gram-negative bacterium, that holds paramount significance within the genus *Klebsiella* of the Enterobacteriaceae family and ranks as one of the more common clinical pathogens (Arato et al., 2021). It is associated with a wide range of infections, including pneumonia, urinary tract infections (UTIs), bacteremia, and liver abscesses (Martin and Bachman, 2018). *K. pneumoniae* can be further classified into two groups based on virulence characteristics: classical *Klebsiella pneumoniae* (cKp) and hypervirulent *Klebsiella pneumoniae* (hvKp) (Li et al., 2019). The cKp primarily causes hospital-acquired infections, with a higher prevalence among immunocompromised patients (Wang et al., 2020). Due to the improper or misuse of antibiotics in clinical settings, cKp has undergone an alarming rate of evolution, as a result, multidrug-resistant (MDR) *K. pneumoniae* that is characterized by its ability to resist three or more antimicrobial agents has emerged as a serious public health concern (Shadkam et al., 2021). On the other hand, hvKp exhibits notably high virulence and has shown a propensity to converge with drug-resistant strains (Hayashi et al., 2021). The hvKp was initially discovered in Taiwan, and it was identified as a major contributor to septic liver abscesses and since then it become a worldwide health threat (Siu et al., 2012). The emergence of drug-resistant hvKp isolates has further complicated the clinical management of infections, necessitating urgent attention (Choby et al., 2020).

Here, we delve into the comparative pathogenesis of drug resistance between cKp and hvKp. We explore the underlying mechanisms contributing to MDR and inspect the increasing prevalence of drug-resistant strains of *K. pneumoniae*. Additionally, we conduct an extensive review of the current status of vaccine developments, with a special focus on potential strategies for targeting both classic and hypervirulent strains of *K. pneumoniae*. By enhancing our understanding of *K. pneumoniae* pathogenesis, factors influencing drug resistance, and the epidemiological trends of *K. pneumoniae* infections, this review may offer valuable insights and provide future directions for addressing the urgent public health challenges posed by this growing *K. pneumoniae* epidemic.

## 2 Pathogenesis of *Klebsiella pneumoniae* infections

### 2.1 Comparison of virulence factors and pathogenesis on cKp and hvKp

The pathogenesis of classical and hypervirulent *Klebsiella pneumoniae* strains vary significantly, particularly in their capsular structure, virulence genes, and iron acquisition strategies (Choby et al., 2020). Hypervirulent strains are distinguished by the production of K1 and K2 capsular types, with the mucoviscosity-associated gene (magA gene) unique to K1 strains. Sialic acid further enhances resistance to phagocytosis. Lipopolysaccharides (LPS) contribute to bacterial spread in the bloodstream and colonization of internal organs by providing resistance to serum killing through O-antigen and inhibiting phagocytosis via core polysaccharides.

Virulence is also increased by biofilm formation, mediated by Type 1 pili (T1P), which enhances phagocytosis through FimH, and Type 3 pili (T3P), which is implicated in lung infections (Choby et al., 2020). Iron acquisition plays a key role in virulence, with siderophores such as enterobactin, salmochelin, yersiniabactin, and aerobactin helping bacteria absorb host iron. While enterobactin is inhibited by the host molecule lipocalin-2, other siderophores evade this inhibition. Specific transporters like *Ferric enterobactin* receptor (FepA), *salmochelin* N (IroN), *yersiniabactin Q* (YbtQ), and *Ferric aerobactin* receptor (IutA) facilitate the uptake of trivalent iron in a siderophore-dependent manner, supporting bacterial survival in iron-limited conditions.Hypervirulent strains of *Klebsiella pneumoniae* carry key virulence factors encoded on a large virulence plasmid known as pLVPK. This plasmid includes the rmpA and rmpA2 genes, which regulate the mucoid phenotype, contributing to the hyperviscosity of these strains. Additionally, several iron acquisition systems are encoded on the same plasmid, enhancing the bacterium’s ability to acquire iron in the host environment, a crucial factor for its survival and virulence. The latest research shows that interpreting *blaSHV* alleles in *Klebsiella pneumoniae* genomes is complex due to various mutations, which can lead to extended-spectrum β-lactamase (ESBL) or β-lactamase inhibitor (BLI) resistance (Choby et al., 2020). The study systematically reviewed the experimental evidence for SHV enzyme function, reclassifying 37 *SHV* alleles and improving the mapping of genotype to phenotype. These findings have been integrated into the Kleborate v2.4.1 tool, enhancing the accuracy of gene classification and aiding in the understanding of antimicrobial resistance mechanisms.

**FIGURE 1:**
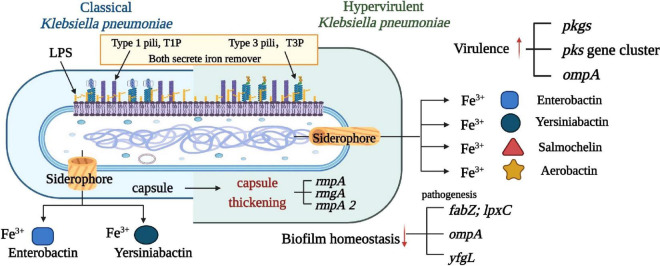
Comparison of virulence factors between classical and highly virulent *K. pneumoniae*. This figure illustrates a comparative analysis of virulence factors found in classical and highly virulent strains of *K. pneumoniae*. Four well-characterized virulence factors are highlighted: capsule, LPS, Pili (types 1 and 3), and siderophore. Notably, the siderophore is a feature shared with other pathogens such as Enterobacter, Salmonella, Yersinia, and Oxytocin. Understanding these distinctions in virulence factors is crucial in elucidating the varying pathogenicity and clinical outcomes associated with different *K. pneumoniae* strains.

**FIGURE 2:**
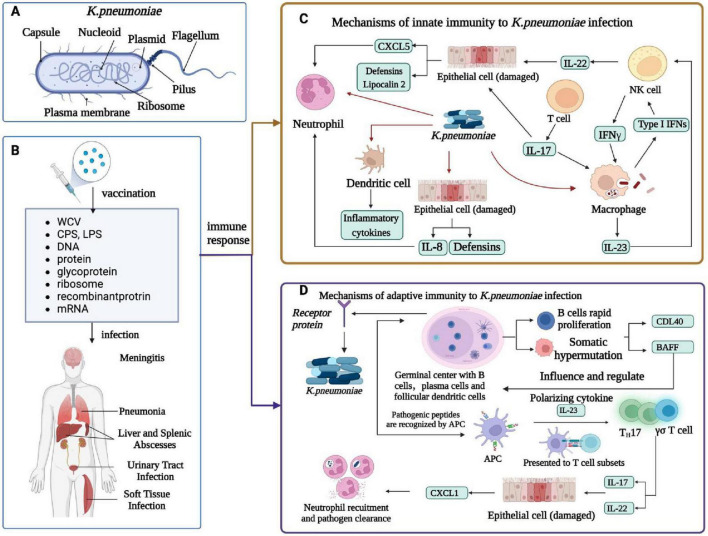
Immune response of *K. pneumoniae* infection. **(A)** Cellular composition of *K. pneumoniae.* **(B)** *K. pneumoniae* infection in humans and related vaccines. *K. pneumoniae* infections include meningitis, pneumonia, liver and spleen abscesses, UTIs, and soft tissue infections. The vaccines against *K. pneumoniae* include whole-cell vaccine, capsular polysaccharide vaccine, LPS -related vaccine, protein vaccine, conjugate vaccine, ribosomal vaccine, reverse vaccine, DNA vaccine, and mRNA vaccine. **(C)** Innate immunity of *K. pneumoniae* infection. *K. pneumoniae* can induce innate immune system activation and interact with neutrophils, macrophages, dendritic cells, and epithelial cells. DC cells produce inflammatory cytokines, macrophages are a major cellular target of IFN γ, NK cells are a major source of IL-22, and IL-17 and IL-22 help in the clearance of *Klebsiella* by regulating the antimicrobial activity of the epithelium; Together they produce CXCL5 and Lipocalin 2, which recruits neutrophil, and epithelial cells produce defensins and IL-8, which recruits neutrophil. The alveolar macrophage produces inflammatory cytokines, IL-23, and type I IFN, and IL-23 activates NK cells to produce cytotoxic effects. **(D)** Adaptive immunity in humans caused by *K. pneumoniae* infection. Antigen-activated germinal center B cells undergo rapid proliferation and somatic hypermutation of their immunoglobulin variable genes, follicular dendritic cell *in vivo* secrete t-cell signals CD40L and b-cell activating factor (Baff) that affect and regulate b-cell survival and differentiation, and b-cell production of antibodies that kill bacteria. The APC recognizes the Antigen-presenting cell peptide and presents it to a subset of T cells in a local lymph node. Apcs release polarized cytokines (for example, IL-23) that can activate Th17 and γδ T cells. These T cell subsets produce IL-17 and IL-22 and stimulate airway epithelial cells expression to mediate chemokines (for example, CXCL1). Activated neutrophils engulf and kill bacteria, thereby enhancing *K. pneumoniae* clearance and local inflammation resolution.

The virulence factors of *K. pneumoniae* are encoded by the genes of the core genome and the accessory genome called chromosomal and plasmid-encoded genes (Wang et al., 2020). The virulence factors identified so far include capsular polysaccharides (CPS), lipopolysaccharide (LPS), siderophore, fimbriae, pilus, plasma membrane, and ribosomes (Wang et al., 2020). The hvKp-specific virulence plasmid and which of the defined virulence factors are located on this genomic element, which is primarily responsible for the hvKp pathotype. Figure 1 in the illustration demonstrates a side-by-side comparison of virulence factors in classical and highly virulent strains of *K. pneumoniae*. It’s worth noting that the siderophore is a feature shared with other pathogens like *Enterobacter*, *Salmonella*, and *Yersinia*. Understanding these distinctions in virulence factors is crucial for comprehending the varying levels of pathogenicity and clinical outcomes associated with different strains of *K. pneumoniae*. Cellular composition included capsule, nucleotide, plasmid, flagellum, pilus, plasma membrane, and ribosome. Figure 2 illustrates the immune response to *K. pneumoniae* infection. It provides a comprehensive view of the cellular composition involved in *K. pneumoniae* infection and the diverse strategies used for immunization, highlighting the key immune players in combating this pathogen.

#### 2.2.1 Capsular polysaccharides

CPS, are the main virulence factor, and their production is controlled by a set of regulatory genes called *rmpA* and *rmpA2.* The cKp strain has acquired in incomplete hvKp-specific virulence plasmid, which does not contain sufficient hvKp-specific genes to confer the hypervirulent pathotype. The presence of CPS protects the bacteria from phagocytosis, antimicrobial peptide activity, and complement-mediated lysis, and also evasion from the host immune response (Opoku-Temeng et al., 2019). Till now, a total of 141 different K antigens have been identified (Namikawa et al., 2019; Patro et al., 2020). The most prevalent capsule types or K antigens in hvKp include K1, K2, K5, K20, K54, and K57 (Russo and Marr, 2019). K antigens such as K1 and K2, are closely related with the ability to simultaneously carry *rmpA*, *magA*, *iroN, sitC*, and aerobactin *aerobactin 3(iuc3)* virulence genes (Russo and Marr, 2019). HvKp contains more CPS than cKp, resulting in a high mucous phenotype and increased virulence of hvKp (Choby et al., 2020). HvKp strains produce supercapsules, which are stronger than typical capsules and potentially play a significant role in the pathogenicity of hvKp (Zhu et al., 2021). Three *rmpA* genes have been identified in hvKp strains, consisting of two plasmid-carried genes and one chromosome gene located in the virulence plasmid pLVPK and play an important role in increasing virulence by regulating the synthesis of CPS (Xu et al., 2021). The strong correlation between *rmpA* genes and high virulence makes it a useful biomarker for the identification of potential hvKp strains (Arato et al., 2021). The K1 biosynthetic gene is a crucial virulence factor found in invasive *K. pneumoniae* strains and it is located on the chromosome, associated with both the high viscosity of the bacteria and resistance to leukocyte phagocytosis (Lee et al., 2017).

**FIGURE 3:**
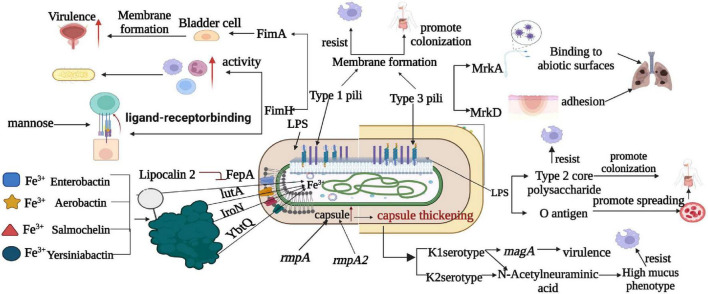
Comparison of pathogenesis mechanism between classical and highly virulent *K. pneumoniae*. This figure provides an insightful comparison of the pathogenesis mechanisms observed in classical and highly virulent *K. pneumoniae* strains. Notable factors and mechanisms include the production of K1 and K2 capsular types, the presence of magA exclusively in K1 strains, and the role of sialic acid in enhancing anti-phagocytic activity. LPS is shown to promote bacterial dissemination in the bloodstream and colonization in internal organs through o-antigen resistance to serum killing and anti-phagocytosis by core polysaccharides. Furthermore, T1P and T3P contribute to strain virulence by forming biofilms, with T1P amplifying phagocytosis via FimH and T3P causing lung infections through various mechanisms. This figure also highlights iron metabolism as a critical factor, with siderophores (Enterocin, Salmonella, Yersinia, and Aerobacteriaceae) aiding in virulence by absorbing host iron. It’s noted that Enterobacter is inhibited by the host molecule lipocalin-2, whereas Yersinia, Salmonella, and aerobactinins are not, and specific transporters (FepA, IroN, YbtQ, and IutA) facilitate the transport of trivalent iron to bacterial cells in a siderophore-dependent manner.

Figure 3 presents a comprehensive comparison of pathogenesis mechanisms between classical and highly virulent strains of *K. pneumoniae*. It sheds light on key factors and mechanisms that differentiate these strains in terms of virulence. Notably, the figure highlights the distinct capsular types K1 and K2, with the exclusive presence of magA in K1 strains, contributing to their virulence. Additionally, the role of sialic acid in enhancing anti-phagocytic activity is illustrated, showcasing its significance in *K. pneumoniae* pathogenesis Furthermore, sialic acid, an essential component of K1 and K2 CPS, plays a key role in the hypermucoviscous phenotype (Zhu et al., 2021). In addition, certain capsulate-related genes such as *wabG*, *uge*, and *ycfM* also play an important role in virulence by contributing to anti-phagocytic activity (Ballén et al., 2021).

#### 2.2.2 Lipopolysaccharide

LPS is composed of three components: the O antigen, the core polysaccharide, and the highly conserved hydrophobic lipid A (Bertani and Ruiz, 2018). The O antigen serves to prevent the binding of complement C1q and C3b to bacteria, thus inhibiting the activation of the complement system pathways. The core polysaccharide aids in resisting phagocytosis by alveolar macrophages. Additionally, lipid A undergoes modifications through various enzymes during transportation, leading to a reduction in inflammatory activation and enhancing the resistance to the host natural innate immunity, particularly against antimicrobial peptides (Raetz et al., 2007).

*K. pneumoniae* possesses a minimum of nine O antigen serotypes, among them O1 is the predominant O antigen serotype in hvKp (Palusiak, 2022). Antigen O1 confers resistance against serum killing and facilitates the dissemination of bacteria into the bloodstream, as well as the colonization of internal organs (Hsieh et al., 2012). For instance, in the mouse model of pneumonia, the LPS of hvKp is not directly associated with lung infection (Shankar-Sinha et al., 2004). However, the presence of its O1 antigen can facilitate bacterial dissemination into the bloodstream, leading to bacteremia and higher mortality rates (Hsieh et al., 2012; Figure 3). In the mouse model of UTIs, the O5 antigen of hvKp contributed to bacterial adhesion to urinary epithelial cells and colonization of the urethra (Shankar-Sinha et al., 2004). Both cKp and hvKp strains possess an intact LPS that offers protection to *K. pneumoniae* against the host immune response (Russo and Marr, 2019).

Figure 3 delves into the involvement of LPS in *K. pneumoniae* pathogenesis, elucidating how LPS promotes bacterial dissemination in the bloodstream and colonization in internal organs. It underscores the resistance of o-antigen to serum killing and the anti-phagocytic properties conferred by core polysaccharides. Moreover, T1P and T3P are shown to play pivotal roles in strain virulence by forming biofilms. T1P amplifies phagocytosis via FimH, while T3P contributes to lung infections through various mechanisms.

#### 2.2.3 Adhesin

Adhesin plays a crucial role in the effective colonization of *K. pneumoniae* infection (Guerra et al., 2022). Among the most reported types are T1P, T3P, KPC, KPF-28, ECP, and CF29K. The type I and III pili of *K. pneumoniae* exhibit robust adhesive activity, enabling them to attach bacteria to the surface of the host cells, leading to entry, replication, and infection (Martin and Bachman, 2018). Type I pili contain various proteins such as FimA, B, C, D, E, and H, and the main structural protein FimA, accounts for more than 95% of the total protein of type I pili (Arato et al., 2021). T1P plays a significant role in *K. pneumoniae* UTIs by facilitating the invasion of bladder cells promoting the formation of biofilms on the surface and also increasing the phagocytosis by macrophages and neutrophils (Clegg and Murphy, 2016). The binding of FimH subunits to the host immune cells augments the activity of these immune cells, leading to increased host clearance of *K. pneumoniae* (Figure 3). The primary structural component of T3P is MrkA, which predominantly binds to non-biological surfaces. Meanwhile, the tip of MrkD primarily binds to the extracellular matrix and functions as a bacterial hair adhesin (Arato et al., 2021). MrkB, MrkC, and MrkE are responsible for assembly and expression regulation, whereas MrkF is also involved in ensuring the surface stability of bacterial pili (Arato et al., 2021). Two modes of *K. pneumoniae* pulmonary infection exist: MrkA binds directly to abiotic surfaces, facilitating *K. pneumoniae* adhesion to endotracheal tubes, while MrkD binds to collagen or extracellular matrix surfaces derived from bronchial cells. Adhesins of microbes are linked to biofilm formation, which in turn enhances the strain’s host defense and antibiotic resistance, thereby increasing bacterial virulence. HvKp has a high rate of acquisition of antimicrobial resistance determinants as compared to cKp, which is the main reason for its resistance to the available antibiotics (Guerra et al., 2022). Recently a research group has identified that biofilm formation in the NTUH-K2044 hvKp strain plays a significant role in bacterial colonization and invasion of the host cell (Shon et al., 2013). This observation suggests that increased biofilm formation is a significant factor contributing to the increased virulence of hvKp. Both cKp and hvKp exhibited the presence of KPC hairs and CF29K, with hvKp showing a higher prevalence of these factors (Di Martino et al., 1995).

#### 2.2.4 Siderophores

Iron metabolism emerges as a critical factor in *K. pneumoniae* pathogenesis, with siderophores such as Enterocin, Salmonella, Yersinia, and Aerobacteriaceae aiding in virulence by facilitating the absorption of host iron. Figure 3 highlights the distinct interactions of host molecule lipocalin-2 with Enterobacter and other pathogens, revealing how certain transporters (FepA, IroN, YbtQ, and IutA) facilitate the transport of trivalent iron to bacterial cells in a siderophore-dependent manner. This comprehensive depiction of pathogenesis mechanisms provides valuable insights into *K. pneumoniae* virulence. Iron is an essential cofactor for bacterial metabolism, which acts as a redox catalyst for oxygen and electron transport processes (Bradley et al., 2020). The greater the bacteria ability to compete with the host for iron acquisition, the faster their growth and metabolism, and the higher their virulence (Sheldon et al., 2016). Iron carriers are low molecular weight proteins that bind to trivalent ions and provide a supply of iron to bacteria. The transport of trivalent iron to bacterial cells relies on a complex family of proteins, including TonB, ExB, and ATP-binding cassette (ABC) transport proteins (Sheldon et al., 2016). Among these, the TonB system in *K. pneumoniae* has been demonstrated to make a significant contribution to bacterial virulence (Miethke and Marahiel, 2007).

Iron carriers bind to ferric ions, forming iron-iron carrier chelates. These chelates then interact with the iron carrier receptor protein on the cell membrane and enter the cell through the ABC transporter protein, facilitating its absorption by bacterial cells (Miethke and Marahiel, 2007). Iron acquisition is an important role for these siderphores, whereas their role in decreasing reactive oxygen species (ROS) production is less clear. The ferric-uptake regulator is a key regulatory protein that controls gene expression in response to iron concentrations. *K. pneumoniae* possesses four major iron carriers, namely enterobactin, salmochelin, yersiniabactin, and aerobactin (Karampatakis et al., 2023; Figure 1). Enterobactin is considered as the primary iron uptake agent and exhibits the highest affinity for iron, while both yersiniabactin and aerobactin indirectly diminish or reduce the bactericidal capacity of host immune cells by inhibiting the production of reactive oxygen species, which of yersiniabactin are found in less than 18% of cKp and 90% of hvKp (Martin and Bachman, 2018). Compared to cKp, hvKp secretes a higher quantity of more potent iron carriers (Choby et al., 2020). HvKp can acquire host iron through four iron carriers to support its metabolism and enhance virulence during infection. Among these four carriers, aerobactin plays a critical role in iron acquisition, growth, and virulence of *K. pneumoniae*, making it a key virulence factor for hvKp (Wang et al., 2020). Salmochelin, yersiniabactin, and aerobactin are more frequently found in hvKp strains than in cKp strains (Russo and Marr, 2019). The three iron carriers, including aerobactin, are not hindered by lipocalin-2 (Figure 3). As a result, strains containing these iron carriers exhibit higher virulence, particularly in the case of aerobactin. Furthermore, the genes that encode iron uptake systems are located adjacent to T6SS-related genes, and it is believed that T6SSs may provide hvKp with a survival advantage (Zhu et al., 2021).

The HmuRSTUV heme/hemoglobin uptake system constitutes a critical mechanism by which *K. pneumoniae* scavenges and utilizes heme and hemoglobin as iron sources (Li et al., 2014). Research elucidate the system’s pivotal role in heme acquisition, bacterial proliferation, and virulence during bloodstream infection (Lan et al., 2023). Their findings reveal that an hmuST allele—based typing scheme exhibits significantly greater allelic diversity in hypervirulent *K. pneumoniae* (hvKP) lineages than in multidrug-resistant (MDR-KP) lineages, thereby demonstrating its potential for distinguishing between hvKP and MDR-KP strains.

## 3 Mechanism of resistance and spreading of multidrug-resistant *Klebsiella pneumoniae*

### 3.1 Resistance mechanism

**FIGURE 4:**
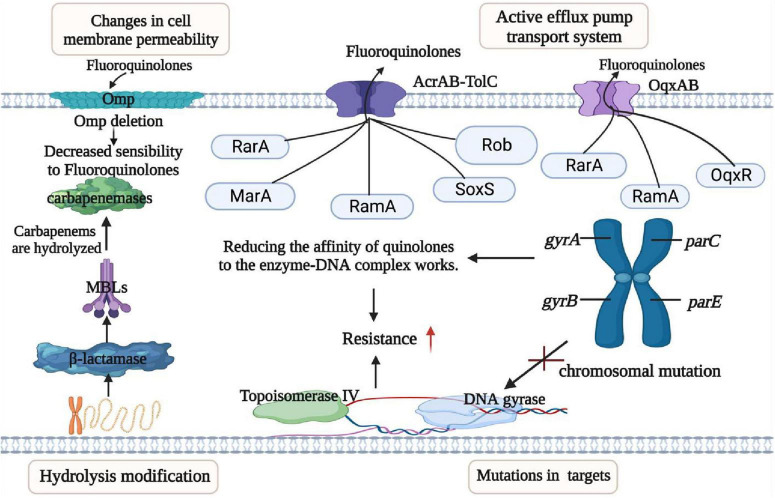
Mechanisms of resistance in *K. pneumoniae*. This figure outlines the resistance mechanisms employed by *K. pneumoniae*, categorized into four main groups: (I) Permeability Reduction: Mutations or deletions, particularly in adventitial channels like porin Omp, lead to decreased permeability of the bacterial cell membrane. (II) Antibiotic Modification: *K. pneumoniae* exhibits resistance through the hydrolytic modification of antibiotics, notably β-lactamases that hydrolyze β-lactam antibiotics. (III) Target Structure Alterations: Resistance is also achieved by mutations and transformation of antibiotic target structures on the bacterial cell surface, reducing the affinity of antibiotic molecules. (IV) Efflux Pump Overexpression: Overexpression of efflux pumps, such as AcraB-TolC and OqxAB, contributes to resistance by reducing antibiotic accumulation within the bacterial cell. Transcriptional regulators like RarA, MarA, RamA, SoxS, Rob, and OqxAB are involved in regulating these efflux pumps. Understanding these resistance mechanisms is crucial for developing effective strategies to combat antibiotic-resistant *K. pneumoniae* infections.

The emergence of MDR *K. pneumoniae* is profoundly impacted by the improper use of broad-spectrum antibiotics, and the mechanisms of resistance exhibit great diversity. These mechanisms include hydrolytic modification of antibiotics, mutation of the antibiotic target site, reduced permeability, and overexpression of efflux pumps (Zhu et al., 2021). Modification of antibiotics by hydrolysis is a major mechanism of resistance, and *K. pneumoniae* can produce extended-spectrum β-lactamases (ESBLs) for the modification of available antibiotics (Zhu et al., 2021). β-lactam hydrolases target β-lactam antibiotics and extend their activity to mannose-binding lectin (MBL) and other carbapenemases. MBLs along with carbapenemases, can hydrolyze a wide range of β-lactam antibiotics, including penicillin, cephalosporins, aminoglutethimide, and carbapenems, leading to the ineffectiveness (Karampatakis et al., 2023; Figure 4). Another mechanism of resistance involves mutations in antibiotic target sites, particularly in the case of quinolones. Quinolone resistance arises from chromosomal mutations occurring in the quinolone resistance-determining region (QRDR) of DNA gyrase and topoisomerase IV. These mutations commonly occur in a localized domain of the GyrA and ParE subunits of gyrase and topoisomerase IV (Hooper and Jacoby, 2016).

Tigecycline sensitivity is reduced by chromosome-mediated modifications of the 30S and 16S ribosomal units, which are the targets of the tigecycline action (Jo and Ko, 2021). Additionally, mutations in *plsC*, *rpsJ*, *trm*, *tet*(A), and *tet*(M) genes also contribute to the decreased sensitivity to tigecycline (Sun et al., 2019). LPS modification systems facilitate the modification of LPS, resulting in reduced negative charge and decreased affinity for mucin, which ultimately leads to resistance (Linkevicius et al., 2013). Mutations in the two-component system PmrAB and PhoPQ, as well as their modulators (e.g., MgrB), can lead to the addition of L-Ara4N and PETN into lipid A within the LPS structure, consequently elevating the minimum inhibitory concentration (MIC) and decrease the susceptibility of microorganisms (Liu X. et al., 2022). Aminoglycoside resistance can arise from target site modifications caused by 16S rRNA methyltransferases (RMT), that modify specific nucleotide residues in the rRNA of the 16S subunit, preventing aminoglycosides from effectively binding to their intended target sites (Lv et al., 2020).

The reduction in permeability is related to the ineffectiveness of antibiotics in entering or crossing the bacterial outer membrane through pore protein channels to reach their sites of action (Vasan et al., 2022). Low intracellular concentration of antibiotics occurs when certain hydrophilic antibiotics, such as β-lactams, tetracyclines, chloramphenicol, and fluoroquinolones, diffuse through the outer membrane’s aqueous channels formed by pore proteins (Omp) (Richter et al., 2017). These porins proteins facilitate the passage of antibiotics, allowing them to traverse into the cell. The quantity, form, and quality of outer membrane pore proteins in various Gram-negative bacteria can be down-regulated or mutated, leading to reduced permeability and increased resistance (Vergalli et al., 2020). This effect may synergistically combine with basal or elevated expression of efflux transport proteins. Resistance to polymyxins in *Salmonella enterica* can be increased through the interaction of the periplasmic protein (YdeI) with the OmpD pore protein regulated by the two-component system of PhoPQ and PmrAB (Nang et al., 2019). Tigecycline resistance mechanism involves the downregulation of the outer membrane pore protein gene, *ompK35*, which leads to altered cell permeability, contributing to resistance (Park et al., 2023; Figure 4). The gene cluster *tmexCD1-toprJ1* carried by plasmid, that encodes a resistant nodal superfamily RND-type efflux pump and responsible for conferring resistance to tetracyclines and also leads to reduced susceptibility to various clinically significant antimicrobial drugs (Lv et al., 2020). Overexpression of multidrug efflux systems belonging to the RND family of multidrug efflux pumps leads to reduced accumulation of antibiotics in the cytoplasm which provides bacteria with sufficient time to replicate and acquire resistance (Duraes et al., 2018). Tigecycline resistance in *K. pneumoniae* is associated with significant roles played by overexpression of the intrinsic chromosomally encoded RND-type efflux pumps AcrAB and OqxAB. These pumps are overexpressed due to mutations in transcriptional regulatory genes (*ramR* and *acrR*) (Lv et al., 2020). Additionally, the insertion of ISAba1 into the AdeS gene results in a truncated soluble AdeS kinase protein being produced through the promoter on ISAba1 which overexpress the AdeABC pump, resulting in tigecycline resistance (Jo and Ko, 2021; Figure 4).

### 3.2 Mechanism of transmission

**FIGURE 5:**
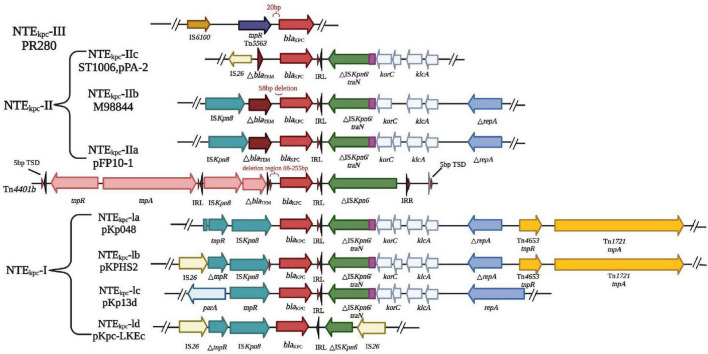
Genetic mobile elements (TN*4401* and NTE_KPC_) contained by *bla*_KPC_. In *K. pneumoniae*, the antibiotic resistance gene *bla*_KPC_ is carried by genetic mobile elements, specifically TN4401 and NTE_KPC_. These elements are classified based on the presence of particular insertion sequences located upstream of *bla*_KPC_. NTE_KPC_ is categorized into three groups: groups: NTE_KPC_-I, without insertion; NTE_KPC_-II, with insertion Δ*bla*_TEM_; NTE_KPC_-III, with insertion TN *5563*/IS *6100*. NTE_KPC_-I: This group lacks upstream insertions and can be further subdivided into AS-IA (e.g., prototype pKP048), -IB (e.g., pKPHS2), -IC (e.g., pKP13D), and -Id (e.g., pKPC-LKEC). This classification depends on the insertion sites of IS26 and the presence of ISkpn8. NTE_KPC_-II: These elements contain an insertion known as Δ*bla*_TEM_. NTE_KPC_-II further divides into -IIa (e.g., pFP10-1), -IIb (identified in strains M9884 and M9988), and -IIc (e.g., PPA-2). These subcategories are determined by variations in the length and deletions associated with △ *bla*_TEM_. Understanding these genetic mobile elements is crucial for unraveling the mechanisms of antibiotic resistance conferred by *bla*_KPC_ in different *K. pneumoniae* strains. These insights are invaluable in the fight against antibiotic resistance in clinical contexts.

There are three main mechanisms by which bacterial resistance genes disseminate among bacterial species; horizontal transfer of transposons, horizontal transfer of plasmids, and spread of clonal populations (Lv et al., 2020). The Tn3-based transposon Tn*4401* is the most common mobile element containing *bla*_KPC_. This transposon includes *bla*_KPC_, Tn3 transposase gene (*tnpA*), Tn3 catabolic enzyme gene (*tnpR*), and two insertion sequences, *Kpn6* and *Kpn*7 (Figure 4). Figure 4 illustrates the resistance mechanisms employed by *K. pneumoniae*. These mechanisms are critical for the bacterium’s survival and defense against antibiotics. The core structure in the horizontal transport of *bla*_KPC–2_ may be involved in IS*Kpn27*-*bla*_KPC–2_-IS*Kpn6* (Zhang F. et al., 2021). The diverse 5bp target site sequence of Tn*4401* supports its high transposon mobility. Figure 5 provides an overview of genetic mobile elements associated with the antibiotic resistance gene *bla*_KPC_ in *K. pneumoniae*. This gene is carried by two primary mobile elements: TN4401 and NTEKPC, which play a significant role in the spread of antibiotic resistance. A common hotspot for Tn*4401* is transposon Tn*1331*, which gives rise to a heterozygous transposon structure (Ramirez et al., 2019). Tn*1331* carries Tn3-like transposase and catabolic enzyme genes (*tnpA* and *tnpR*), aminoglycoside modifying enzyme genes *aac(6′)-Ib* and *aad*A1, as well as the β-lactamase genes *bla*_OXA–9_ and *bla*_TEM–1_. The *bla*_KPC_ gene was also found in non-Tn*4401* mobile elements (NTE) (Figure 5). NTE_KPC_ is mainly present in non-ST258 *K. pneumoniae* or other non-*K. pneumoniae* species; whereas, *bla*_KPC_ in epidemic ST258 *K. pneumoniae* strains is exclusively carried on Tn4401.

**FIGURE 6:**
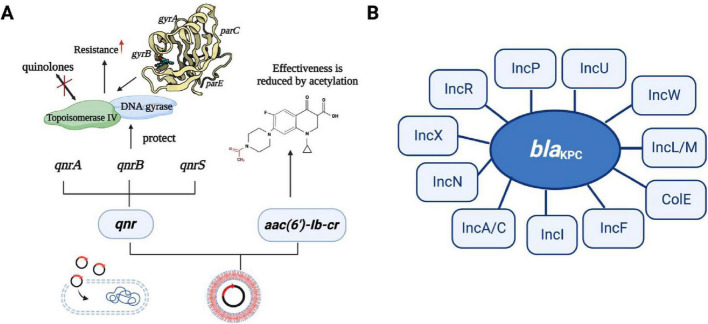
The transmission mechanism of resistance in *K. pneumoniae*. **(A)** The spread of resistance genes. *qnr* gene and aminoglycoside acetyltransferase variant gene [AAC(6′)-Ib-c] are commonly found in multiple resistance plasmids. Three quinolone resistance mediators are QNR protein, which can be divided into QnrA, QnrB, and QnrS, they protect against quinolone inhibition leading to resistance to the target enzyme encoding DNA helicase and topoisomerase IV; whereas a variant of the aminoglycoside-modified acetyltransferase AAC(6′)-Ib acetylates certain quinolones, reducing antibiotic activity leading to resistance, and then completes dissemination by plasmid transfer into host cells. **(B)** The type of plasmid backbone containing *bla*_KPC_. The plasmids with different types of *bla*_KPC_ included IncF, IncI, IncA/C, IncN, IncX, IncR, IncP, IncU, IncW, IncL/M, and Cole plasmids.

Resistance genes can be transferred horizontally between bacteria via plasmids (Su et al., 2020). CRKP strains exhibit multi-drug resistance due to the presence of *bla*_KPC_ on large plasmids. These plasmids also carry other resistance determinants, conferring resistance to a range of antibiotics, including aminoglycosides, quinazolines, methicillin, sulfonamides, and tetracyclines (Ying et al., 2020). These resistance genes are highly transmissible, contributing to the dissemination of multi-drug resistance among CRKP strains. Mobile or transposable factors are consistently linked with *qnr* genes, especially ISCR1 and IS*26*. The *qnr* genes are typically found in multi-resistant plasmids alongside other resistance determinants. These plasmids encoded resistance by producing Qnr proteins, which shield target enzymes from the action of quinolones (Amereh et al., 2023). There are three known quinolone resistance mediators, namely Qnr proteins: QnrA, QnrB, and QnrS, which protect against quinolone inhibition leading to resistance to the target enzyme encoding DNA helicase and topoisomerase IV; whereas a variant of the aminoglycoside-modified acetyltransferase AAC(6′)-Ib acetylates certain quinolones, reduces antibiotic activity leading to resistance, and then completes dissemination by plasmid transfer into host cells (Hooper and Jacoby, 2015; Figure 6A). The carbapenemase gene transfer is linked with two IS*26* elements of class I integrons. These elements are transferred at the interbacterial level by transmitting the resistance gene to the adapter conjugate (Ying et al., 2020). Gene *bla*_KPC_ is found on various types of plasmids (Figure 6B), and ColE-type plasmids, ranges in size from 10 to 300 kb (Chen et al., 2014). The genetic analysis of six *bla*_KPC_-bearing plasmids from different incompatibility groups revealed a common structure among them. The *Escherichia coli (E. coli)* strains carrying *bla*_KPC_, small Col-like plasmids are the second most prevalent type of plasmid and are commonly associated with KPC producers (Chen et al., 2014). These small vectors have been demonstrated to play a significant role in facilitating the interspecies spread of the *qnr* genes, which confers resistance to fluoroquinolones (Chen et al., 2014). Plasmid-mediated polymyxin/colistin resistance is attributed to the *mcr* family genes, which encode a group of phosphoethanolamine transferases responsible for facilitating the horizontal transfer of resistance to polymyxins (Wang et al., 2016; Wareth and Neubauer, 2021). These genes are found in different plasmids with diverse backbones and their size ranges from 58 to 251Kb (Gao et al., 2016). Common plasmids with *mcr-1* can be used as helper plasmids to further promote plasmid propagation-mediated tigecycline resistance (Karki et al., 2021). These interconnected evolutionary fine-tunings may contribute to maintaining or enhancing the bacterial fitness of these prevalent clones and aid in the dissemination of clonal populations.

### 4 Immune response against *Klebsiella pneumoniae* and vaccine development

*K. pneumoniae* can elicit robust innate immune system activation and interact with neutrophils, macrophages, dendritic cells, and epithelial cells (Gonzalez-Ferrer et al., 2021). DC cells produce inflammatory cytokines, while macrophages are a major cellular target of IFN-γ (Van Elssen et al., 2010). NK cells are a major source of IL-22, and IL-17 to help in the clearance of *Klebsiella* by regulating the antimicrobial activity of the epithelium tissue. Collectively, these epithelium produces CXCL5 and Lipocalin 2, which in turn recruits neutrophils, and epithelial cells with the help of defensins and IL-8 (Ahn and Prince, 2020). The alveolar macrophage produces inflammatory cytokines IL-23 and type I IFN, and IL-23 which activates NK cells to produce cytotoxic effects (Wei et al., 2023; Figure 2C). *K. pneumoniae* can also induce adaptive immunity in humans, leading to the activation of germinal center B cells. These cells rapidly proliferate and undergo somatic hypermutation in their immunoglobulin variable genes. Follicular dendritic cells *in vivo* secrete T-cell signals CD40L and B-cell activating factor (Baff) that affect and regulate B-cell survival, differentiation, and B-cell production of antibodies that kill bacteria (Chen et al., 2019; Tang et al., 2021). The antigen-present cells recognize the antigen-presenting cell peptide and present it to a subset of T cells in a local lymph node (Roche and Furuta, 2015). The antigen-present cells release polarized cytokines (i.e., IL-23) that can activate Th17 and γδ T cells. These T cell subsets produce IL-17 and IL-22 and stimulate airway epithelial cells expression to mediate chemokines (for example, CXCL1)(Cai et al., 2010). Activated neutrophils engulf and kill bacteria, thereby enhancing *K. pneumoniae* clearance and local inflammation resolution (Figure 2D). To combat the infections caused by *K. pneumoniae*, a series of related vaccines have been developed, including whole-cell vaccine, capsular polysaccharide vaccine, LPS-related vaccine, protein vaccine, conjugate vaccine, ribosomal vaccine, reverse vaccine, DNA vaccine, and mRNA vaccine (Ranjbarian et al., 2023; Figure 2B).

### 4.1 Polysaccharide vaccines

#### 4.1.1 Capsular polysaccharide vaccines

CPS are the main protective antigens and virulence factors of *K. pneumoniae*. The high immunogenicity and surface exposure of the capsule make it a viable target antigen for bacterial vaccines, as it enables effective vaccination. Despite the effectiveness of capsular oligosaccharide conjugate vaccines against infections caused by enveloped pathogens, there is currently no available vaccine that specifically targets *K. pneumoniae* (Yang et al., 2019). Due to the wide structural variation of CPS, 79 serotypes of CPS have been identified primarily (Follador et al., 2016; Hsu et al., 2016). The structural variability and geographic distribution of serotypes pose limitations to the potential coverage of capsular oligosaccharide conjugate vaccines. In 2017, Seeberger et al. (2017) developed a semi-synthetic vaccine known as CRM197-1, which is synthesized from CPS and related sequences. CRM197-1 vaccine induced high levels of antibodies in mice and rabbits, also these antibodies showed cross-reactivity with natural CPS. However, further research is required to investigate and evaluate the efficacy of this vaccine. Initial investigations involving monovalent and hexavalent CPS vaccines revealed that these vaccines exhibited excellent tolerance in healthy individuals. Based on this, a 24-valent CPS vaccine has been developed and several clinical trials have been conducted (Choi et al., 2019; Table 1).

**TABLE 1:** Progress toward the development of *Klebsiella* vaccines.

Vaccine classification	Antigen	Animal model	Injection method	Protective efficacy (Y/N)	Measurement	References
Whole cell vaccines	*K. pneumoniae tonB* deletion mutant	Mouse	IP^a^	Y	Evaluated IgG level	(Green et al., 1970)
	Live attenuated *K. pneumoniae*	Mouse	IN or PO	ND^b^	Evaluated cytokines and IgA levels	(Hackett and Marcus, 1970)
	Acetone-dried *K. pneumoniae*	Rabbit	IV	ND	Evaluated antibody level	(Woodworth et al., 2018)
	Mixed bacterial vaccine (MBV)	Human	PO	Y	Evaluated IgG and IgA level	(Nikolova et al., 2009)
Capsular polysaccharide (CPs) vaccines	12 CPSs^c^	Mouse	IP	Y	ND	(Hsieh et al., 2008)
	K1, K36, K44, and K Cross^d^	Mouse	IP	Y	ND	(Alaubydi et al., 2018)
	24 CPSs^e^	Human	SC	ND	Evaluated IgG	(Riottot and Fournier, 1981)
	K2, K3, K10, K21, K30, and K55	Human	SC	Y	Evaluated IgG level	(Jones and Roe, 1984)
	K1, K2	Human	SC	Y	Evaluated IgG and IgM level	(Feldman et al., 2019)
O polysaccharide (OPs) vaccines	*Klebsiella* O1 LPS into liposomes	Rat	IM	Y^b^	ND	(Donta et al., 1996)
	*Klebsiella* O1 LPS	Mouse	IM, IT, or IN	Y^b^	Evaluated IgG level	(Zigterman et al., 1985)
	Neisseria meningitidis outer membrane protein (Omp) complex of *Klebsiella* O1 LPS	Rat	IP	Y^b^	Reduce the survival rate of *K. pneumoniae*	(Cross et al., 2001)
Protein vaccine	*K. pneumoniae* cytotoxin	Mouse and rabbit	ID	Y	Evaluated IgG level in mice and baby rabbit born from immunized mother	(Wang et al., 2017)
	Purified type 3 fimbriae of *K. pneumoniae*	Mouse	IP	Y	Evaluated IgG level in mice	(Babu et al., 2017)
	Recombinant AK36 protein comprised of antigens from OmpA and OmpK36	Mouse	SC	Y	Evaluated IgG, IgM, and IgA levels	(Serushago et al., 1989)
	Eight surface proteins^f^	Mouse	SC	Y	ND	(Li et al., 2010)
	Siderophore receptor protein	Cattle	SC	ND	Reduced *K. pneumoniae* mastitis and increased milKProduction	(Singh and Sharma, 2001)
	CusC, OmpN, FepB, ZnuA, RNase HI, TehB	Mouse	SC	ND	ND	(Mehmood et al., 2020)
Conjugate vaccines	Octasaccharide derived from CPS (K11) coupled to bovine serum albumin	Mouse	IP	ND	Evaluated IgG level	(Edelman et al., 1994)
	O1 Ops linked to tetanus toxoid	Rat	ID	Y^b^	Alveolar macrophage activation	(Granström et al., 1988)
	Hexasaccharide 1 coupled to diphtheria toxin mutant	Mouse and rabbit	SC	N^b^	Evaluated IgG, and IgM levels	(Cryz et al., 1986)
	*K. pneumoniae* OPS (O1, O2, O3, O5) linked to *P. aeruginosa* (PA) flagellin protein (FlaA, FlaB)	Mouse	IM	Y	Evaluated IgG level	(Seeberger et al., 2017)
	K1 and K2 CPS conjugate vaccine	Mouse	IM	Y	The number of bacteria alive in serum	(Lin et al., 2022)
DNA vaccine	Vector pVAX1 expressing OmpA or ompk36	Mouse	ID or IM	N	Evaluated cytokines and IgG levels	(Klipstein et al., 1983)
MAPS vaccine	Four *K. pneumoniae* OPS (01, 02, 03, 05) and MrkA^e^	Mouse	IM	Y	Evaluated IgG level	(Chhibber et al., 2004)
Vesicle vaccine	EVs^g^	Mouse	IP	Y	Evaluated cytokines and IgG levels	(Hussein et al., 2018)

^a^IN, Intranasal; PO, oral; SC, subcutaneous; IP, intraperitoneal; IV, Intravenous; SL, sublingual; ID, intradermal; IM, intramuscular; IT, intratracheal.

^b^ND, Not determined. Reduced organ burden, but did not test survival.

^c^K1, K2, K3, K15, K20, K35, K36, K44, K50, K63, K70, and K74 antigens.

^d^K Cross refers to cross-reactive CPS.

^e^K2, K3, K5, K9, K10, K15, K16, K17, K18, K21, K22, K25, K28, K30, K35, K43, K52, K53, K55, K60, K61, K62,K63, and K64 antigens.

^f^GlpQ, Glycerophosphodiester phosphodiesterase, RecX, recombination regulator; YhiN, oxidoreductase; NirB, nitrite reductase subunit; YfhM, hypothetical protein; RecO, DNA repair protein RecO; GlnH, glutamine ABC transporter periplasmic protein; MrdA, penicillin-binding protein 2.

^g^This vaccine also includes Pseudomonas OPS and protein.

#### 4.1.2 Lipopolysaccharides-related vaccines

O-antigen fraction of the LPS hinders the phagocytosis of the bacterium by macrophages and neutrophils, as well as the production of cytokines (Gyorfy et al., 2013). Bacterial O-antigens are categorized into nine serotypes, wherein serotypes O1, O2, and O3 are responsible for the majority of infections (Choi et al., 2019). LPS is capable of triggering immune protection against *K. pneumoniae* infection in mice, and O antigen-specific antibodies can be applied for the treatment or prevention of *K. pneumoniae* infection. Additionally, studies have indicated the robust immunogenicity of LPS and its capacity to exhibit cross-reactivity with other Gram-negative bacilli, thereby highlighting its potential as a promising vaccine candidate (Zariri and van der Ley, 2015). The potent endotoxicity of LPS represents a major barrier to its direct use as a vaccine antigen: at high doses, LPS can provoke severe tissue and organ injury, culminating in septic shock (Miller et al., 2024). Such pronounced endotoxic responses preclude its clinical application due to the risk of serious adverse effects. To overcome these limitations, structural modification and derivative development have been pursued. For example, chemical conversion of LPS into monophosphoryl lipid A (MPL) dramatically attenuates its toxicity while preserving the majority of its immunomodulatory activity (Casella and Mitchell, 2008). In addition, bioengineering non-pathogenic bacterial strains to produce LPS variants with altered TLR4–MD2 recognition and cytokine-induction profiles offers a promising avenue for the generation of low-toxicity, high-efficacy LPS-based vaccines (Maeshima and Fernandez, 2013).

### 4.2 Protein vaccines

Proteins are antigens and due to their ability to elicit an immune response, they can be used as vaccine candidates. The immunogenic proteins of *K. pneumoniae* are the external membrane proteins (EMPs) and pilin protein (Zhu et al., 2021). EMPs and type III pilus have been utilized in the development of experimental vaccines (Xie et al., 2020). Five EMPs have been used in a mice model and identified that these vaccines increase the production of antigen-specific IgG, IgG1, and IgG2a antibodies. In infection models of *K. pneumoniae*, it was observed that only Kpn_Omp001, Kpn_Omp002, and Kpn_Omp005 were capable of eliciting protective immune responses (Zhu et al., 2021). Furthermore, these protective effects were accompanied by distinct immune responses induced by the *K. pneumoniae* OmpA. Based on this, it can be inferred that Kpn_Omp001, Kpn_Omp002, and Kpn_Omp005 represent three promising candidate antigens that may trigger Th1, Th2, and Th17 immune responses. These antigens hold the potential to be developed into multivalent vaccines that are effective across different serotypes, offering protection against *K. pneumoniae* infections (Zhang B. Z. et al., 2021).

In a recent study, an integrated informatics and genome analysis pipeline was employed to rationally design a multiepitope subunit vaccine targeting four high-risk serotypes (Wang et al., 2022). The investigators initially conducted *in silico* epitope prediction and antigenicity screening, followed by molecular docking to characterize the interactions of the construct with Toll-like receptors (TLRs). Subsequently, molecular dynamics simulations were performed to evaluate the conformational stability of the vaccine in a simulated physiological fluid environment. This comprehensive computational framework enabled the identification of candidate epitopes with optimal immunogenicity, strong TLR binding affinities, and robust structural integrity under near physiological conditions.

### 4.3 Conjugate vaccines

Conjugate vaccines achieve long-lasting immune protective effects through a chemical process involving the covalent binding of polysaccharide antigens to carrier proteins, resulting in the formation of glycoproteins (Wantuch and Rosen, 2023). This covalent linkage enables the vaccine to elicit a stronger and more sustained immune response compared to using polysaccharide antigens alone. Among bacterial vaccines, glycoconjugate vaccines, which involve the binding of bacterial polysaccharides to carrier proteins, have emerged as one of the most successful approaches to date (Liu et al., 2016). The immunogenicity of polysaccharides is commonly enhanced by protein carrier conjugates. The conjugate vaccines, which utilized the pilin protein of *K. pneumoniae* and the O-specific polysaccharide (OPS) of *E. coli* serotype K12, exhibited significantly increased antibody titers against OPS semi-antigens and bacterial cilia as carrier proteins. Currently, a glycoconjugate vaccine is in development, comprising four *K. pneumoniae* OPS serotypes conjugated to *Pseudomonas aeruginosa* (*P. aeruginosa*) flagellin (Hegerle et al., 2018). The OPS vaccine has shown immunogenicity in animal models that received passively transferred antibodies induced by the OPS vaccine exhibited protection against systemic cKp infection. However, it is more suitable for targeting cKp strains due to the masking effect of the podoconjugate polysaccharide found in highly virulent isolates, which can obscure the OPS antigen. Concurrently, a nano-coupled vaccine has been developed utilizing the OPS with galactose repeats from *K. pneumoniae* (Peng et al., 2021). This OPS-based nanocouple vaccine shows promising potential as a potent antigen within a nanovaccine preparation platform (Liu et al., 2016). Additionally, a bioconjugation approach has been developed, by utilizing glycoengineered *E. coli* that expresses K1 and K2 antigens of *K. pneumoniae* under the modulation of *rmpA*. This innovative method triggers a serotype-specific IgG response in mice, effectively protecting them from infection caused by K1 and K2 strains of *K. pneumoniae* (Liu et al., 2016). The coupling of OPS with PA Fla and PA glycoconjugate vaccines leads to a formulation that generates antibody titers against four *K. pneumoniae* OPS types and two PA Fla antigens. This enhanced formulation can provide broad protection against multiple *K. pneumoniae* OPS types and *P. aeruginosa* flagellin (Liu et al., 2016). The development of conjugate vaccines encounters certain limitations, mainly attributed to the utilization of a single type of carrier protein and the high production costs involved, to restrict the flexibility and cost-effectiveness of the vaccine development process.

### 4.4 Reverse vaccines

Reverse vaccinology involves the analysis of a pathogens genome sequencing to identify antigenic determinants. Subsequently, high-throughput cloning, expression, and purification techniques are applied to obtain recombinant proteins of the antigenic determinants. The candidate antigens are then subjected to *in vivo* and *in vitro* evaluations to screen for the protective antigens, which can be further utilized in vaccine development. This approach allows for more targeted and efficient screening of potential vaccine candidates. Compared to traditional vaccine research methods, reverse vaccines offer greater convenience, broader scope, and enhanced safety. Employed antigenomic techniques have been applied to screen 169 antigenic proteins of *K. pneumoniae* from clinical diagnoses (Lundberg et al., 2013). Subsequently, through *in vitro* analysis and murine infection models, eight novel proteins capable of inducing active immunity and providing protection were identified (Mehmood et al., 2020). This highlights the potential of reverse vaccinology in identifying promising vaccine candidates efficiently and effectively. These eight newly identified proteins are expected to be utilized in the preparation of *K. pneumoniae* vaccines. Reverse vaccinology paves the way for a novel approach to vaccine development, enabling the prediction and discovery of previously undiscovered immunogens. This approach is anticipated to play an increasingly significant role in future vaccine development endeavors.

### 4.5 mRNA vaccine

In recent years, mRNA vaccine technology has emerged as a promising avenue in the fight against MDR *K. pneumoniae* infections. These innovative vaccines are tailored to target specific genes associated with drug resistance or the production of virulence factors in *K. pneumoniae* (Khalid and Poh, 2023).

The concept behind mRNA vaccines is ingenious. They are designed to encode precise segments of these critical genes, stimulating the immune system to mount a robust response against the encoded proteins. For example, mRNA vaccines can effectively zero in on β-lactamase genes, provoking an immune response that directly counters the β-lactamase enzyme’s activity. This, in turn, diminishes the enzymes ability to deactivate antibiotics, thereby enhancing the antibiotics effectiveness.

• β-lactamase genes: These enzymes play a pivotal role in antibiotic resistance by deactivating β-lactams. mRNA vaccines encode specific segments of genes responsible for β-lactamase expression, provoking an immune response that directly counters the β-lactamase enzymes activity. This strategy enhances the efficacy of antibiotics.
• Efflux pump: Efflux pumps actively expel antibiotics from bacterial cells, rendering them less effective. mRNA vaccines can hinder the production of efflux pumps, promoting higher antibiotic retention within the bacterial cell.
• Carbapenemase: Carbapenemases are enzymes that degrade carbapenem antibiotics. mRNA vaccines induce an immune response that neutralizes the carbapenemase enzyme, thereby improving the efficacy of carbapenem antibiotics.
• Virulence factor genes: Virulence factors are crucial for *K. pneumoniae*’s ability to evade the host immune response and colonize tissues. mRNA vaccines can be carefully designed to combat these genes, stimulating the production of antibodies that neutralize key virulence factors like capsules, LPS, fimbriae, or siderophores. This approach significantly impairs the bacterium’s ability to establish infections (Van der Put et al., 2023).

By harnessing the body immune response mechanisms, mRNA vaccines represent a highly promising strategy for addressing antibiotic-resistant bacteria. They hold immense potential in the prevention and control of MDR *K. pneumoniae* infections by precisely targeting drug resistance and virulence factors.

However, it’s crucial to emphasize that rigorous testing and validation in both preclinical and clinical studies are imperative to establish the safety and efficacy of these mRNA vaccines. These steps are critical in our collective efforts to combat antibiotic resistance effectively and manage MDR *K. pneumoniae* infections in a rapidly evolving medical landscape. Developing mRNA vaccines for *K. pneumoniae* presents several scientific challenges that need to be addressed for successful vaccine implementation. These challenges include:

• Target Antigen Selection: Identifying the most suitable antigens from *K. pneumoniae* that induce a robust immune response while avoiding potential autoimmune reactions is challenging. These antigens must be highly specific to *K. pneumoniae* and play a critical role in its virulence or survival.
• Antigen Diversity: *K. pneumoniae* exhibits considerable genetic diversity, even within strains of the same species. Designing an mRNA vaccine that covers this genetic diversity to ensure broad protection against different *K. pneumoniae* strains is complex.
• Delivery and Stability: mRNA vaccines require efficient delivery methods to reach the target cells and initiate an immune response. Additionally, ensuring the stability of mRNA vaccines during storage and transportation at various temperatures can be challenging.
• Immune Evasion Mechanisms: *K. pneumoniae* employs various immune evasion mechanisms, such as capsule formation and efflux pumps. Overcoming these mechanisms to ensure the vaccine effectively stimulates an immune response against *K. pneumoniae* is a significant hurdle.
• Safety Concerns: mRNA vaccines must be carefully designed to minimize potential adverse effects. Balancing vaccine efficacy with safety is a crucial challenge.
• Clinical Trials: Conducting comprehensive clinical trials to evaluate the safety and efficacy of mRNA vaccines for *K. pneumoniae* is a complex and resource-intensive process, requiring substantial time and investment.
• Long-Term Immunity: Ensuring that the vaccine confers long-term immunity against *K. pneumoniae* infections and provides protection even as *K. pneumoniae* evolves is a critical challenge.
• Regulatory Approval: Meeting the rigorous regulatory standards for vaccine approval is a lengthy and demanding process that must be successfully navigated (Liu et al., 2023).

Tackling these challenges requires collaboration between researchers, clinicians, and regulatory agencies. Continued advancements in mRNA vaccine technology and a deep understanding of *K. pneumoniae* biology and pathogenesis will be pivotal in developing effective vaccines against this MDR pathogen (Hegerle et al., 2018).

### 4.6 Other types of vaccines

Vaccine development has undergone a shift from using whole-cell vaccines comprised of intact microorganisms expressing multiple antigens to subunit or non-cellular vaccines containing well-defined, purified antigens. This transition has resulted in reduced reactogenicity of the vaccine and enabled the production of repeatable and stable vaccines (Mba et al., 2023). Bacterially secreted outer membrane vesicle (OMV), known as extracellular vesicles (EVs), are the most widely used antimicrobial membrane nanomaterials. Bacterial bionanobacterial vesicle (BBV) vaccines offer a unique advantage with their dual function of inducing both bacterial-specific humoral and cellular immune responses. These vaccines have demonstrated the capability to enhance animal survival rates, reduce lung inflammation, and lower bacterial burden (Opoku-Temeng et al., 2019). The establishment of BBV vaccine platforms has greatly optimized the technology for developing vaccines against MDR *K. pneumoniae* (Hirabayashi et al., 2021). In addition, microencapsulated vaccines, multi-epitope vaccines, and DNA vaccines can elicit specific immune responses against *K. pneumoniae* (Assoni et al., 2021). Also, iron carriers and membrane receptor cells have been explored as potential vaccine candidates, which play a crucial role in the virulence of the pathogen and are uniquely positioned to facilitate rapid recognition by the host immune system (Gorden et al., 2018). Another vaccine candidate involves live attenuated strains of D-glutamate-deficient mutants, which lack a key structural component of the bacterial cell wall (Cabral et al., 2017). The vaccine effectively induces a protective immune response against systemic infections caused by various *K. pneumoniae* strains.

## 5 Epidemiological aspect of *Klebsiella pneumoniae* infection and distribution of drug-resistant *Klebsiella pneumoniae* strains

MDR *K. pneumoniae* bacterial species are resistant to three or more antibiotics such as resistance toward a class of aminoglycosides and cephalosporins (Shadkam et al., 2021). *K. pneumoniae* strains are highly resistant to β-lactams, quinolones, and aminoglycosides (Table 2) but sensitive to carbapenems (Wang et al., 2020; Table 3). However, after the use of carbapenems and fluoroquinolones for infections caused by *K. pneumoniae*, more than 400 acquired resistance genes have been identified in the genome of *K. pneumoniae* (Wyres and Holt, 2018). *K. pneumoniae* nosocomial infections are caused by *K. pneumoniae* carbapenemase-producing bacteria all over the world (Zhan et al., 2021).

**TABLE 2:** Antibacterial drugs effective against *K. pneumoniae* infections.

Types	Pharmaceuticals	Usage (adults with normal liver and kidney function)	Approved by FDA	Approved by CFDA	Production companies
Penicillin	Amoxicillin and potassium clavulanate	1.2 g IV drip 1/8 h	√	√	SANDOZ, teva, Aurobindo, etc.
	Ampicillin and Sulbactam	1.5–3 g IV drip 1/6 h	×	×	/
	Piperacillin sodium and sulbactam sodium	3.375 g IV drip 1/6 h	×	×	/
Cephalosporins	Cefazolin	1 g IV drip 1/12 h	√	√	Baxter, GSK, BD Bioscience, etc.
	Cefuroxime	1.5 g IV drip 1/12 h	√	√	SUN Pharma, GSK, BD Bioscience, etc.
	Cefotaxime	1 g IV drip 2/day	√	√	Hospira, Fresenius Kabi, BD Bioscience, etc.
	Ceftriaxone	1–2 g IV drip 1/day	√	√	Roche, SANDOZ, BD Bioscience, etc.
	Ceftazidime	1 g IV drip 2/day	√	√	Baxter, GSK, Hospira, etc.
	Cefepime	1–2 g IV drip 1/12 h	√	√	Baxter, GSK, BD Bioscience, etc.
	Ceftazidime-avibactam	2.5 g IV drip 1/8 h	×	√	QILU PHARMACEUTICAL
Cephamycins	Cefoxitin	1–2 g IV drip 1/6–8 h	√	√	Mylan, BD Bioscience, etc.
Aminoglycosides	Amikacin	Adjust usage according to renal function and body weight	√	√	Apothecon Inc., div Bristol Myers Squibb, Hikma, etc.
Aminoglycosides	Tobramycin	Adjust usage according to renal function and body weight	√	√	Novartis, Hospira, teva, etc.
	Gentamicin		√	√	Novartis, Hospira, Baxter, etc.
Tetracyclines	Tigecycline	first dose 100 mg, then 50 mg/12 h	√	√	SANDOZ, Merck, Apotex Corp., etc.
Polypeptides	Polymyxin	500,000 units, 2 times/day	√	√	Pfizer, Novartis, Sandoz, etc.
Nitrofurantoin	/	100 mg PO 1/12 h	√	√	Sun Pharma, Watson Lab, Merck, etc.
Carbapenems	Meropenem	1 g IV drip 1/8 h	√	√	Pfizer, BD Bioscience, Sandoz, etc.
	Imipenem	500 mg IV drip 1/6 h	√	√	Merck, Hospira, ACS Dobfar Spa, etc.
	Imipenem and cilastatin sodium	1.25 g IV drip 1/6 h	√	**×**	Merck, Hospira, ACS Dobfar Spa, etc.
	Ertapenem	1 g IV drip 1 day	√	√	Merck, ACS Dobfar Spa, etc.
Fluoroquinolones	Ciprofloxacin	400 mg IV drip 1/12 h	√	√	Pure Chemistry, TCI, etc.
	Levofloxacin	750 mg IV drip 1/12 h	√	√	Macklin, Merck, TCI, etc.
	Moxifloxacin	400 mg IV drip 1/12 h	√	√	Macklin, Matrix Scientific, TCI, etc.

**TABLE 3:** Antibacterial drugs against drug-resistant *K. pneumoniae.*

Application	Type	Name of commodity	Usage	Approved by FDA	Approved by CFDA	Production companies	Introduction
Extended-spectrum β-lactamase (ESBLs)	Carbapenems	Imipenem	IM/VD	√	√	Merck, Hospira, ACS DOBFAR SPA, etc.	Status: Increasing detection rate year by year causes of resistance: hydrolyzed penicillin, broad-spectrum and ultra-broad-spectrum cephalosporins, and β-lactamases of monocyclic β-lactams.
		Meropenem	IV/VD	√	√	Pfizer, BD Bioscience, SANDOZ, etc.	
	Cephalosporins	Ceftazidime-avibactam	IV	×	√	QILU pharmaceutical	
	Cephamycins	Cefoxitin	IM/IV	√	×	Mylan, BD Bioscience, etc.	
Cephalosporinase resistance	Carbapenems	Meropenem	IV/VD	√	√	Pfizer, BD Bioscience, SANDOZ, etc.	Susceptibility to resistance of *K. pneumoniae* or *E. coli* to third-generation cephalosporins or enzyme inhibitors
	Cephalosporins	Ceftazidime-avibactam	IV	×	√	QILU Pharmaceutical.	
Carbapenem resistance	Tetracyclines	Tigecycline	VD	√	√	SANDOZ, Merck, etc.	Hydrolysis of almost all β-lactam antibiotics including carbapenem antibiotics
	Cephalosporins	Ceftazidime-avibactam	IV	×	√	QILU Pharmaceutical	
	Polypeptides	Polymyxin	PO	√	√	Pfizer, Novartis, SANDOZ, etc.	

Carbapenem-resistant *Klebsiella pneumoniae* (CRKP) infections pose a serious threat to solid organ transplantation (Pagani et al., 2021) and the overall prevalence of early infection in renal transplant recipients was 36.1% and the prevalence of early CRKP infection was 10.4% (Zhang F. et al., 2021). The distribution of individual resistant bacteria is uneven (Elgendy et al., 2018), and KPC-2 and KPC-3 are the main prevalent enzymes mediating carbapenem resistance in *K. pneumoniae* (Yahav et al., 2020). In China, KPC is predominantly prevalent in KPC-2 (Zhang et al., 2018), whereas in other countries such as Europe and the USA, it turns into both KPC-2 and KPC-3. With *K. pneumoniae* ST11 being a widely disseminated high-risk clonal lineage, highly virulent carbapenem-resistant *K. pneumoniae* (hv-CRKP) has attracted increasing attention in recent years (Marques et al., 2019). OXA-48-like carbapenemases are the most common class or subtype of carbapenemases in Enterobacteriaceae identified in North Africa and the Middle East (Pitout et al., 2019).

The proportion of *K. pneumoniae* carrying highly virulent genes has shown a substantial increase compared to previous studies. For instance, the detection and isolation rate of isolation of CRKP in Chinese hospitals has steadily risen annually from 2016 to 2020 (Liu C. et al., 2022). Among the nasopharyngeal swab cultures performed from adults, 11.5% of the isolates were identified as *K. pneumoniae*, with 77.5% of them being hvKp positive (Lin et al., 2015). In New York, USA, CRKP cases declined steadily over the 2016–2020 period, but reversed this trend in the second half of 2021 and early 2022, when the number of cases occurring increased markedly and the incidence of hospitalized cases followed a similar trend (Lee et al., 2023). Additionally, resistance analysis revealed an escalating trend in the rate of resistance to all common antimicrobial drugs among *K. pneumoniae* isolates (Lynch et al., 2021). Among the collected strains, the resistance rate to β-lactamase inhibitors was significantly lower than that of penicillin and cephalosporins without enzyme inhibitors. However, despite this lower resistance, the detection rate of carbapenem-resistant Enterobacteriaceae, especially CRKP, has been increasing annually due to their widespread clinical use. As a consequence, CRKP has emerged as a significant cause of multi-drug-resistant bacterial infections worldwide. A report released by China’s Antimicrobial Resistance Surveillance System stated that the detection rate of CRKP has shown a continuous increase from 6.4% in 2014 to 10.9% in 2019.^1^

In China, ST11 is one of the most predominant clones of hospital-acquired carbapenem-resistant *K. pneumoniae* (HA-CRKP) in many provinces (Liao et al., 2020; Zhan et al., 2021). ST11 CRKP can develop resistance to antimicrobial drugs and carry virulence plasmids to survive in the host (Jin et al., 2021). The highly virulent encoding plasmid binds to the ST11 *bla*_KPC–2_-carrying CRKP strain, making it a highly virulent resistant compound phenotype. In the case of ST11 CR-hvKp infection, ST11 KPC-2-producing CRKP isolated from the patient was more virulent due to the acquisition of pLVPK-like virulence plasmids (Gu et al., 2018). Pathological analysis showed that 80% of CR-hvKp strains in China belonged to ST11 and KPC-2 producing types (Zhang et al., 2020), along with other resistant genes like NDM (Liu Z. et al., 2019), VIM (Dong et al., 2019), and OXA (Shu et al., 2019) have been reported in CR-hvKp. A strain of *K. pneumoniae-producing* NDM-1 and KPC-2 was isolated from a burn infection in China, and several carbapenemase-producing CR-hvKp have been identified (Liu Y. et al., 2019). Besides China, ST11 *K. pneumoniae* strains have also been identified in Portugal, Germany, Spain, Italy, Switzerland, Taiwan, Singapore, and Japan from clinical isolates (Marques et al., 2019). The combination phenotype of high virulence and multi-drug resistance is becoming more prevalent for *K. pneumoniae*, resulting in more serious public health problems.

## 6 Future research prospectives

**FIGURE 7:**
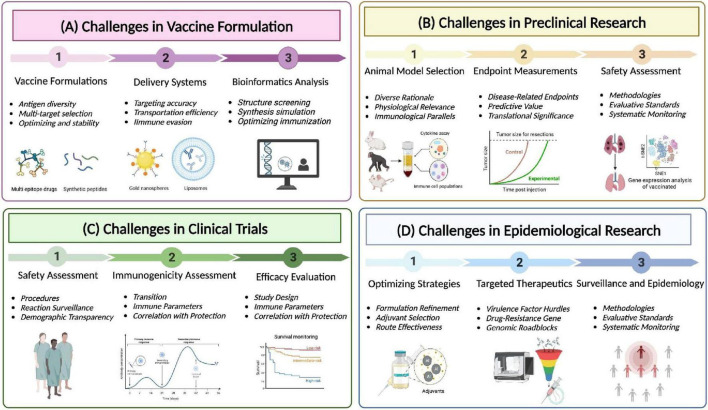
Challenges and advances in multidrug-resistant *Klebsiella pneumoniae* vaccination. **(A)** Challenges in vaccine formulation. Fully leveraging the potential of bioinformatics and bioengineering, develop novel vaccine formulations and delivery systems, and utilize computer-aided design to maximize the response to the threat of multidrug-resistant Klebsiella pneumoniae to human health. **(B)** Preclinical trials: Using mouse, rabbit, and primate animal models to evaluate the efficacy of vaccines to prevent and treat diseases and identify potential side effects. **(C)** Clinical trials: After successful preclinical studies, the vaccines enter the clinical trial stage, and the safety and immunogenicity of the vaccines are comprehensively evaluated through efficacy evaluation system. **(D)** Epidemiology Research: Collect data on the effectiveness of vaccines in reducing or treating human infection rates or severity, and use this data to optimize traditional drugs by combining adjuvants and new targets.

Clinical isolates of *K. pneumoniae* are increasingly developing higher levels of antimicrobial resistance. The emergence of MDR strains in animal hosts and the food chain may eventually pose a threat to humans (Wareth and Neubauer, 2021). Antibiotics remain the primary treatment option for *K. pneumoniae* infections (Opoku-Temeng et al., 2019), and managing hvKp infections requires adequate source control and aggressive antibiotic therapy (Choby et al., 2020). Combination antibiotic therapy is more effective than monotherapy, and novel antimicrobial agents such as polystyrene as an antimicrobial agent are promising candidates against pneumonia infection caused by MDR *K. pneumoniae* (Lou et al., 2018). Immunization strategies are optimized via adapting strategies based on emerging scientific knowledge and refining formulation (Figure 7). Application of novel materials, specifically antibiotic potentiators, have been investigated as a solution against *K. pneumoniae* biofilms (De Oliveira and Franco, 2020). Unfortunately, up to this point, there are no effective strategies to completely eradicate biofilms. Therefore, there is an urgent need to enhance our understanding of various aspects of biofilm formation, organization, communication, and antimicrobial resistance. The high degree of antigenic variation in *K. pneumoniae* makes the development of novel vaccines extremely important. Genomic approaches are crucial for identifying new virulence factors and drug-resistance genes (Figure 7). Furthermore, bioinformatics tools and techniques are utilized to prioritize novel proteins with potential for vaccine development: Copper-silver efflux system Emp (CusC), Outer membrane pore protein (OmpN), Fe enterobacterin transporter protein substrate binding protein (FepB), Zinc transporter protein substrate binding protein (ZnuA), Ribonuclease HI, Tellurite-resistant methyltransferase (TheB), and two uncharacterized hypothetical proteins (WP_002918223 and WP_002892366)(Mehmood et al., 2020). Highly immunogenic T and B cell-specific epitopes of *K. pneumoniae* FepA were identified using a computer prediction tool. The tool successfully pinpointed T (class I and II) and B (linear and conformational) epitopes of FepA, paving the way for potential future vaccine development studies. Notably, a final epitope peptide containing both T and B epitopes was discovered, adding further promise to the potential vaccine candidate (Nemati et al., 2021).

While mRNA vaccines hold great promise, their development for MDR *K. pneumoniae* requires rigorous testing and validation. Detailed information is comprehensively delineated in Figure 7, encompassing preclinical testing, clinical trials, and the future trajectory of research and clinical investigations for vaccines targeting in MDR *K. pneumoniae*. Preclinical studies in animal models can help evaluate the efficacy and safety of mRNA vaccines targeting specific virulence factors and drug-resistance genes. Furthermore, clinical trials are essential to assess vaccine’s immunogenicity and potential adverse effects in humans. In addition, mRNA vaccines targeting virulence factors and drug-resistance genes in MDR *K. pneumoniae* represent a promising avenue for addressing the challenges posed by drug-resistant infections. By eliciting specific and targeted immune responses, these vaccines have the potential to enhance the control and prevention of MDR *K. pneumoniae*, contributing to improved public health outcomes and reduced antibiotic resistance. Continuous monitoring and epidemiological research are indispensable for identifying prevalent strains, resistance patterns, and geographical variations. The primary objective of long-term safety monitoring resides in the detection of rare adverse events or delayed effects. Nevertheless, further research and clinical investigations are important to fully realize the potential of mRNA vaccines in combating MDR *K. pneumoniae* infections.

## 7 Conclusion

This review has provided a comprehensive examination of the epidemiological status of MDR *K. pneumoniae*, as well as in-depth insights into the epidemiological characteristics and pathogenic mechanisms of hvKp. Furthermore, it has offered a concise overview of the current advancements in vaccine development, shedding light on their potential utility as prophylactic and therapeutic measures. However, it is unmistakable that there exists a substantial research and control gap in our understanding and management of *K. pneumoniae* infections. There is an urgent need for further research to uncover the determinants of virulence and drug resistance within these strains. Additionally, more extensive genetic genealogy information, deeper comprehension of transmission mechanisms, the development of highly effective diagnostic methods, the identification of promising antimicrobial therapeutic targets, the discovery of novel antimicrobial agents, and the accelerated progress of vaccine development are all imperative. Addressing these critical areas of investigation is paramount for controlling the dissemination of MDR *K. pneumoniae* and hvKp strains, reducing the associated morbidity and mortality, and mitigating the severity of the public health crisis linked to these infections. Collaboration among researchers, healthcare professionals, and public health authorities is indispensable in meeting these challenges and safeguarding public health effectively.

## Footnotes

1
https://www.chinets.com/
